# Maternal Ethanol Exposure Acutely Elevates Src Family Kinase Activity in the Fetal Cortex

**DOI:** 10.1007/s12035-021-02467-x

**Published:** 2021-07-16

**Authors:** Dandan Wang, Brian W. Howell, Eric C. Olson

**Affiliations:** 1 Department of Neuroscience and Physiology, SUNY Upstate Medical University, 505 Irving Ave, Syracuse, NY 13210 USA; 2 Developmental Exposure to Alcohol Research Center (DEARC), Binghamton University, Binghamton, NY 13902 USA

**Keywords:** Fetal alcohol syndrome disorder, Cortical development, Src kinases, Dab1, Dendritogenesis, Cofilin

## Abstract

Fetal alcohol syndrome (FAS) is characterized by disrupted fetal brain development and postnatal cognitive impairment. The targets of alcohol are diverse, and it is not clear whether there are common underlying molecular mechanisms producing these disruptions. Prior work established that acute ethanol exposure causes a transient increase in tyrosine phosphorylation of multiple proteins in cultured embryonic cortical cells. In this study, we show that a similar tyrosine phosphorylation transient occurs in the fetal brain after maternal dosing with ethanol. Using phospho-specific antibodies and immunohistochemistry, we mapped regions of highest tyrosine phosphorylation in the fetal cerebral cortex and found that areas of dendritic and axonal growth showed elevated tyrosine phosphorylation 10 min after maternal ethanol exposure. These were also areas of Src expression and Src family kinase (SFK) activation loop phosphorylation (pY416) expression. Importantly, maternal pretreatment with the SFK inhibitor dasatinib completely prevents both the pY416 increase and the tyrosine phosphorylation response. The phosphorylation response was observed in the perisomatic region and neurites of immature migrating and differentiating primary neurons. Importantly, the initial phosphotyrosine transient (~ 30 min) targets both Src and Dab1, two critical elements in Reelin signaling, a pathway required for normal cortical development. This initial phosphorylation response is followed by sustained reduction in Ser3 phosphorylation of n-cofilin, a critical actin severing protein and an identified downstream effector of Reelin signaling. This biochemical disruption is associated with sustained reduction of F-actin content and disrupted Golgi apparatus morphology in developing cortical neurons. The finding outlines a model in which the initial activation of SFKs by ethanol has the potential to disrupt multiple developmentally important signaling systems for several hours after maternal exposure.

**Supplementary Information:**

The online version contains supplementary material available at 10.1007/s12035-021-02467-x.

## Introduction

The CDC estimates that 0.2–1.5 per 1000 live births are children with FASD, a syndrome characterized by disrupted fetal brain development and postnatal intellectual disability (ID) [1, 2]. Disrupted connectivity including altered dendritic structure, axonal pathfinding, and white matter tracts are common findings in FAS and are thought to be major contributors to ID [3–6]. However, the cellular and biological targets of alcohol are diverse, and it is not clear whether there are common underlying molecular mechanisms producing these disruptions. Identification of common molecular mechanism(s) would enable a deeper understanding of this disorder, inform studies of genetic susceptibilities, and possibly identify molecular targets for neuroprotective strategies.

This study extends our recent finding that ethanol exposure leads to SFK activation, inappropriate tyrosine phosphorylation in cultured primary neurons [7]. One consequence of the disruption is that the Reelin signaling pathway can no longer be activated in the presence of ethanol [7]. The Reelin-Dab1 signaling is absolutely required for proper cortical development [8], and genetic disruptions of this pathway are associated with disorganized cortical architecture and severe intellectual disability in humans [9]. Reelin is a secreted ligand [10, 11] that orchestrates correct neuronal positioning and dendritic development in many regions of the central nervous system including the cortex, hippocampus, and cerebellum. Complete absence of Reelin [10], the receptors [12], the essential adaptor protein Dab1 [13], or the Src family kinases (SFKs) Src and Fyn [14] leads to similar histological disruptions in the central nervous system (CNS). In humans, a lack of Reelin causes severe ID, ataxia, and mild epilepsy [9].

In prior studies, we observed similarities between ethanol-exposed and Reelin signaling–deficient embryonic neurons including disrupted cellular orientation, primary dendrite numbers, and Golgi compaction [15, 16]. Because our explant multiphoton imaging studies revealed a rapid alteration in dendritic filopodial behavior in response to acute ethanol exposure [7], we focused on biochemical events that occur within the first few minutes after ethanol exposure. This approach led to the discovery of transient increase in general tyrosine phosphorylation levels 10 min following ethanol exposure. This increase is blocked by the Src family kinase (SFK) inhibitors PP2 and SKI-1, but not an inhibitor of Abl tyrosine kinase [7], thereby pinpointing SFKs in the response. The transient increase is followed by a sustained period during which the pathway itself can no longer be activated by in vitro application of the ligand, Reelin. Thus, ethanol appears to disrupt SFK and Reelin signaling in vitro.

In this study, we show that a similar disruption of SFK-dependent phosphorylation occurs in the fetal brain after maternal ethanol exposure. The robust nature of the ethanol-induced tyrosine phosphorylation response allowed us to use immunohistochemistry (IHC) to map changes of phosphorylated tyrosine content in fetal brain sections. The areas of maximal change include the marginal zone (MZ) where Reelin-dependent dendritogenesis occurs and the intermediate zone (IZ) that contains the forming white matter tracts. These areas also correlate with areas of high Src protein expression and Src activation (pY416 activation loop immunosignal). We find that maternal ethanol exposure first causes aberrant Src activation and Dab1 phosphorylation followed by sustained n-cofilin dephosphorylation and Golgi apparatus disruption. This suggests that maternal ethanol exposure may significantly impair SFK and Reelin signaling in the fetal brain.

## Material and Methods

### Animals

All animal procedures were performed in accordance with the Institutional Animal Care and Use Committee of SUNY Upstate Medical University. C57BL/6J mice (Jackson Laboratory) were used in this study. The day of plug discovery is considered embryonic day 0 (E0). Pregnant C57BL/6J were housed in a temperature-controlled room with a 12:12-h light-dark cycle. Intraperitoneal injections of pregnant dams were performed on E15 with either control phosphate-buffered saline (PBS) or PBS/ethanol to achieve 4 g ethanol/kg bodyweight, equivalent to 0.5% v/v. In some experiments, the Src family kinase inhibitor dasatinib (20 mg/kg bodyweight) was injected 30 min prior to ethanol or control injections. Animals were sacrificed 10, 30, 120, and 240 min after injection. Individual embryonic brains were rapidly isolated. Half of the brain was drop fixed in a 4% paraformaldehyde (PFA) PBS solution and then cryoprotected in 30% sucrose before cryostat sectioning for later histological analysis. The dorsal cortex of the other half brain was dissected for subsequent western blot analysis.

### Blood Ethanol Concentration and Fetal Amniotic Ethanol Concentration

Tail blood was collected in heparin coated tubes 10 min prior to the ethanol injections to establish a baseline, and then blood was taken at 10 min, 30 min, 1 h, 2 h, and 4 h after injection. Amniotic fluid was collected immediately after the injected dam had been sacrificed and the uterus exposed. A microsyringe was used to puncture the uterine wall and amniotic sac. A small volume of amniotic fluid was then withdrawn from the amniotic sac for subsequent analysis. The maternal blood and fetal amniotic fluid samples were then centrifuged at 2000 rpm for 15 min and stored at – 80 °C prior to analysis with an Analox Alcohol Analyzer (Analox Instruments, Lunenburg, MA).

### Immunohistochemistry

The 30 μm coronal cryosections were applied to glass slides and allowed to dry at room temperature overnight. Tissue sections were washed in phosphate-buffered saline (PBS) for 15 min and then blocked in 5% donkey serum, 1% BSA, and 0.2% Triton X-100 for 1 h at room temperature and incubated with primary antibody at 4 °C overnight. Mouse anti-pY99 (1:1000, Santa Cruz), rabbit anti-MAP2 (1:200, Protein Tech Co.), rabbit anti-pY416 (1:200, Cell Signaling), rabbit anti-pan Src antibody (1:200, Santa Cruz), rabbit anti-Dcx (1:200, [17], rabbit anti-Tbr1 (1:200, Abcam), rabbit anti-GAD67 (1:200, Millipore), and mouse anti-GM130 (1:200, BD) were used as primary antibodies. Donkey anti-species IgG conjugated with Alexa 488, Alexa 555 or Alexa 647, and Alexa 555-conjugated phalloidin were used as secondary antibodies. Hoechst 33342 (2 μg/ml, Molecular Probes) was used to visualize individual cell nuclei. Images were collected with a Zeiss LSM780 laser scanning confocal microscope (Advanced Fluorescence Imaging Core, SUNY Upstate Medical University). To compare the tyrosine phosphorylation response after different treatment, the average pY99 signal intensity was collected and normalized to the MAP2 immunosignal intensity and then compared among different treated group.

### Cortical Cultures and Treatments

Cortical tissue (E15) was dissociated using 0.25% trypsin at 37 °C for 20 min. A 10:10 buffer (10% trypsin inhibitor and 10% Bovine Serum Albumin, both from Sigma) was used to neutralize trypsin. Neurons were resuspended in Neurobasal medium supplemented with 2% B27, 1% GlutaMAX, and 1% penicillin/streptomycin (all from Invitrogen) and plated onto PDL-coated glass coverslips in 24-well plates. After 3 day in vitro (DIV), cultures were exposed to ethanol (400 mg/dL, 0.5% v/v) or PBS and then fixed for subsequent immunocytochemistry. For western blot analysis, cortical cultures were plated on PDL-coated 12-well tissue culture plates at a final concentration of 2 × 10^6^ cells/ml and then cultured for another 72 h. Lysates were collected after treatment with ethanol (400 mg/dL, 0.5% v/v) or H_2_O.

### Western Blot Analysis

Both adult and E15 embryonic dorsal cortex and primary cortical neuron lysates were collected in radio immunoprecipitation assay (RIPA) buffer (50 mM Tris-HCl; 1% NP-40; 0.25% Na-deoxycholate; 150 mM NaCl; 1 mM EDTA, pH 7.4) with protease inhibitors (Protease Inhibitor Cocktail, Sigma) and phosphatase inhibitors (1 mM Na_3_VO_4_; 1 mM NaF) on DIV 3. All samples were loaded onto 10% SDS-PAGE gels and transferred to 0.45 μm PVDF Immobilon®-FL membranes (EMD Millipore). After incubation with Odyssey blocking buffer (LI-COR Biosciences) for 1 h at room temperature, the membranes were incubated with the following primary antibodies overnight at 4 °C. The primary antibodies were anti-pY99 (1:1000, Santa Cruz), anti-pY416 (1:1000, Invitrogen), anti-pan Src antibody (1:1000, Millipore), anti-Dab1 (1:1000, Sigma), anti-p-Cofilin (Ser3, 1:1000, Invitrogen), anti-Cofilin (1:1000, SantaCruz) and anti-GAPDH (1:2000, UBPBio) were used as loading controls. Appropriate secondary antibodies IRDye® 800CW and IRDye® 680RD (LI-COR Biosciences) were used, and membranes were scanned using the Odyssey® CLx system (LI-COR Biosciences).

### Ex Utero Electroporation and Explant Cultures

Explant preparation and ex utero electroporation were performed on E13 embryos using 0.8 mg/ml of pCAG-GFP construct [15]. Hemispheres were dissected and cultured medial side down on 3 μm pore size, collagen-coated, polytetrafluoroethylene filters (Transwell-COL, Corning) in DMEM-F12 media plus GlutaMAX and supplemented with 1% G5, 2% B27, and 1% penicillin/streptomycin (all from Invitrogen). Explant cultures were maintained in a high oxygen environment (95/5% O_2_/CO_2_) at 37 °C for 48 h before ethanol or control treatments. Explants from both control and ethanol groups were drop fixed in 4% PFA for subsequent histological analysis.

### Golgi Morphology Measurement

To investigate the effect of ethanol on Golgi complex continuity and morphology, we used the Dcx-dsRed^14Qlu/J^ transgenic mouse that expresses dsRed fluorescent protein from a Doublecortin (Dcx) promoter element [18]. The mouse provides genetic identification of developing cortical neurons. The number of puncta and the proximal to distal Golgi length were measured within dsRed^+^ neurons by tracing the GM130 immunosignal through the imaged z-series of the cell. Differentiating neurons were identified as those cells with soma located in the cortical plate and a leading process contacting the marginal zone, whereas migrating neurons were identified as those cells with migrating morphology and a leading process that did not contact the MZ.

### Experimental Design and Statistical Analysis

Graphing and statistical analyses were performed in GraphPad Prism 7. A minimum of two embryos from at least three dams were analyzed for each experiment in each condition. Data are presented as the mean ± SEM. Experiments are designed to determine differences between two groups (control vs. ethanol exposed) using the Student’s t-test. For densitometery and time responses involving multiple measurements, one or two-way ANOVA with post hoc Bonferroni tests was used.

## Results

### Ethanol Increases Tyrosine Phosphorylation After Maternal Exposure

In our previous study, we found that phosphotyrosine levels in lysates of primary cortical cultures derived from Swiss Webster (SW) embryos was transient and largely absent after 30 min of continuous ethanol exposure, a timeline that was paralleled by the in vivo response. To further explore the in vivo response, we used C57Bl6/J mice, a commonly used background used for genetic manipulation and for FAS studies. To provide precise temporal control of the exposure, pregnant dams received an intraperitoneal (i.p.) injection of ethanol (4 g/kg predicted to achieve ~ 400 mg/dL) or PBS on E15. The ethanol dose is within the range of ethanol exposures that have been used in alcohol abuse studies [19, 20] [21] and corresponds to blood ethanol concentration (BECs) that can be found in human subjects with alcohol abuse disorder [22, 23]. The injected dams typically remained conscious during the dosing period, and maternal BEC measurements reached ~ 250 mg/dL at 60 min post injection declining to 116 mg/dL by 4 h after injection. In contrast, amniotic fluid EC achieved a concentration of 430 mg/dL, 10 min after injection, the predicted value based on the injection and similar to 400 mg/dL dose used in our prior in vitro study [7]. Four hours after injection the amniotic fluid showed an EC of 120 mg/dL, equivalent to maternal BEC (see Supplemental Figure). The dams were then euthanized, the embryos were removed, and the embryonic brain was split sagitally: One hemi-brain was drop fixed in 4% paraformaldehyde for subsequent IHC analyses, and the remaining hemi-brain was further dissected in ice cold saline to isolate dorsal neocortex, which was then used to generate lysates for western blot analyses.

**Fig. 1:**
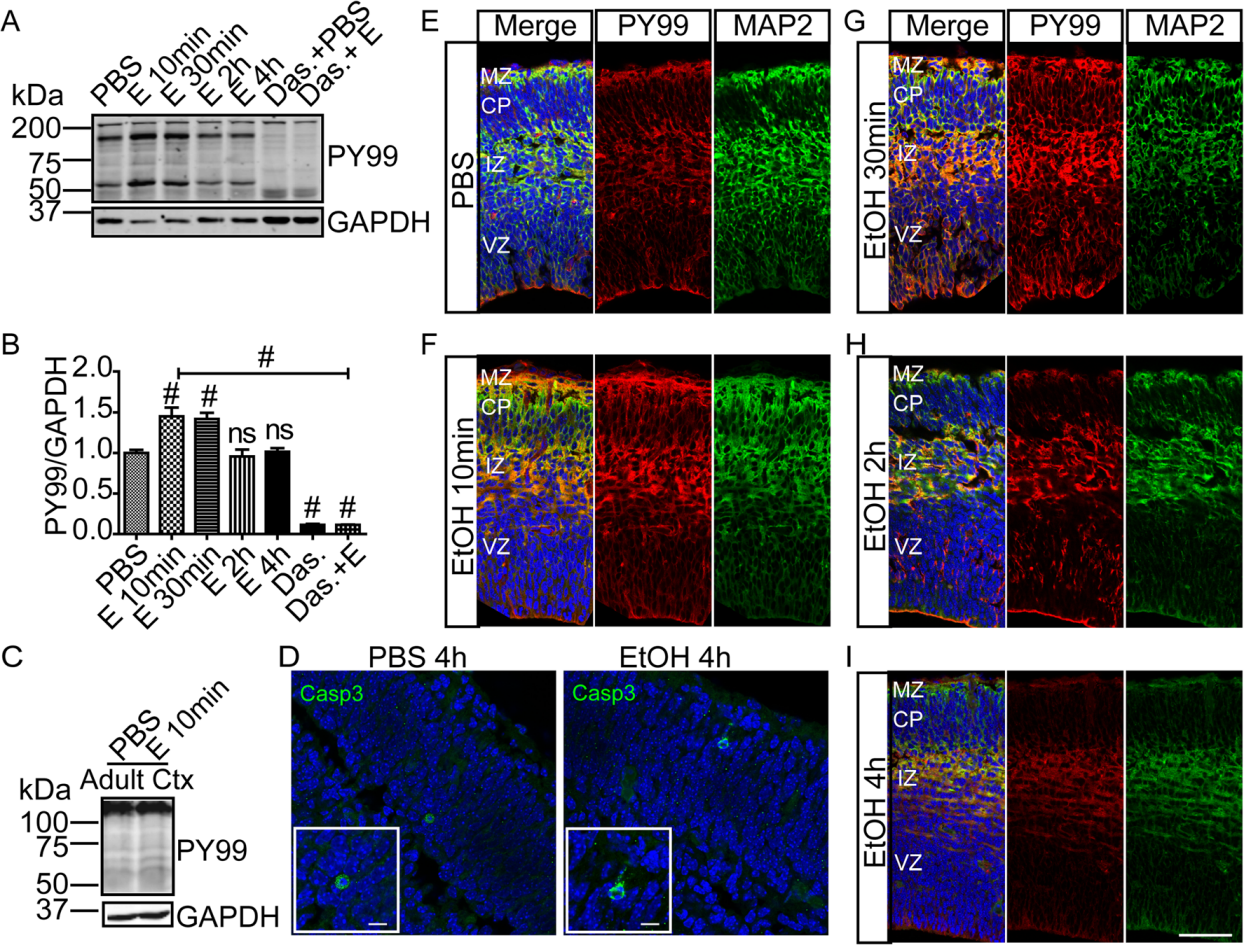
Maternal ethanol exposure induces transient tyrosine phosphorylation in vivo. **A** Western blot detection of tyrosine phosphorylation level in E15 embryonic cortical lysate after PBS or 10, 30, 120, and 240 min of maternal ethanol exposure (i.p., 4 g/kg). **B** Quantification of anti-phosphotyrosine (pY99) signal shows a sustained increase in fetal cortical phosphotyrosine levels for 30 min after maternal exposure that is completely blocked by maternal pretreatment with the SFK inhibitor dasatinib (20 mg/kg). Densitometric values are expressed as total pY99/GAPDH and then normalized to the control (PBS-injected) group for each timepoint. **C** Western blot detection of tyrosine phosphorylation level in maternal cortical lysate after 10 min of PBS or ethanol exposure. **D** Cleaved caspase 3 staining of E15 embryonic brain after 4 h of PBS or ethanol injection revealed few immunopositive cells in either condition. Scale bar, 10 μm, **E–I** pY99 and anti-MAP2 immunostaining in E15 cerebral cortex after **E** PBS or **F** 10 min, **G** 30 min, **H** 120 min, and **I** 240 min of ethanol exposure. Prominent increases in pY99 signal are observed at 10 and 30 min in marginal zone (MZ), the area of apical dendrite projection, and the intermediate zone (IZ), the area of axonal projection. Cortical plate (CP), ventricular zone (VZ). Scale bar, 100 μm. Statistical determination by one-way ANOVA followed by Bonferroni’s post hoc tests between groups: ^#^*p* < 0.001

Elevated phosphotyrosine (pY99) levels were observed in fetal cortical lysates at 10 min after maternal exposure and persisted for at least 30 min before returning to baseline by 4 h (Fig. 1 A and B). The ~ 1.5-fold increase in total pY99 signal was in the same direction but smaller in magnitude than the ~ 3.5-fold increase in pY99 signal observed with SW. However, the C57Bl6/J response was sustained and was detected at 30 min, whereas the SW response had returned to baseline by this time. This difference in response profile appears to be intrinsic to the neuronal cells as the same approximate timeline differences were observed in dissociated cell cultures prepared from the two strains [7] (Fig. 5 A and B). The acute ethanol response may be a strain-specific difference like those observed with rodent ethanol consumption [24] and fetal response to exposure [25]. Interestingly, a tyrosine phosphorylation response was not observed in lysates prepared from the maternal cortex (Fig. 1C), suggesting that embryonic neural tissue is particularly responsive to exposure. Cell death has been associated with ethanol exposure [26]. Although we found no reduction of cell viability with 400 mg/dL dose in culture [7], sections of fetal brains were examined for evidence of cell death using cleaved caspase 3 immunohistochemistry. Caspase 3^+^ cells were infrequently detected in either condition, and no difference in positive cells was identified between conditions 4 h after exposure (Fig. 1D). This finding shows that C57Bl6/J embryos, like SW embryos, demonstrate a rapid tyrosine phosphorylation response to acute maternal ethanol exposure.

To determine whether the in vivo fetal cortical response depended on SFK activation, we pretreated pregnant dams with dasatinib (BMS-354825). Dasatinib is an FDA-approved inhibitor of SFKs, with additional inhibition of BCR-ABL [27] [28], and is used for treatment of chronic myelogenous leukemia [29]. Dasatinib (final concentration of 20 mg /kg) was i.p. injected into the dam 30 min before ethanol or PBS treatment (also i.p. injected). Dasatinib pretreatment completely blocked ethanol-induced increases in tyrosine phosphorylation (Fig. 1 A and B). In addition, dasatinib pretreatment followed by PBS injection lowered pY99 content below baseline (untreated) suggesting that the pY99 signal in the neocortex at E15 may largely be dependent on ongoing SFKs activity.

### Elevated Phosphotyrosine Levels Are Found in Areas of Active Neurite Growth

**Fig. 2:**
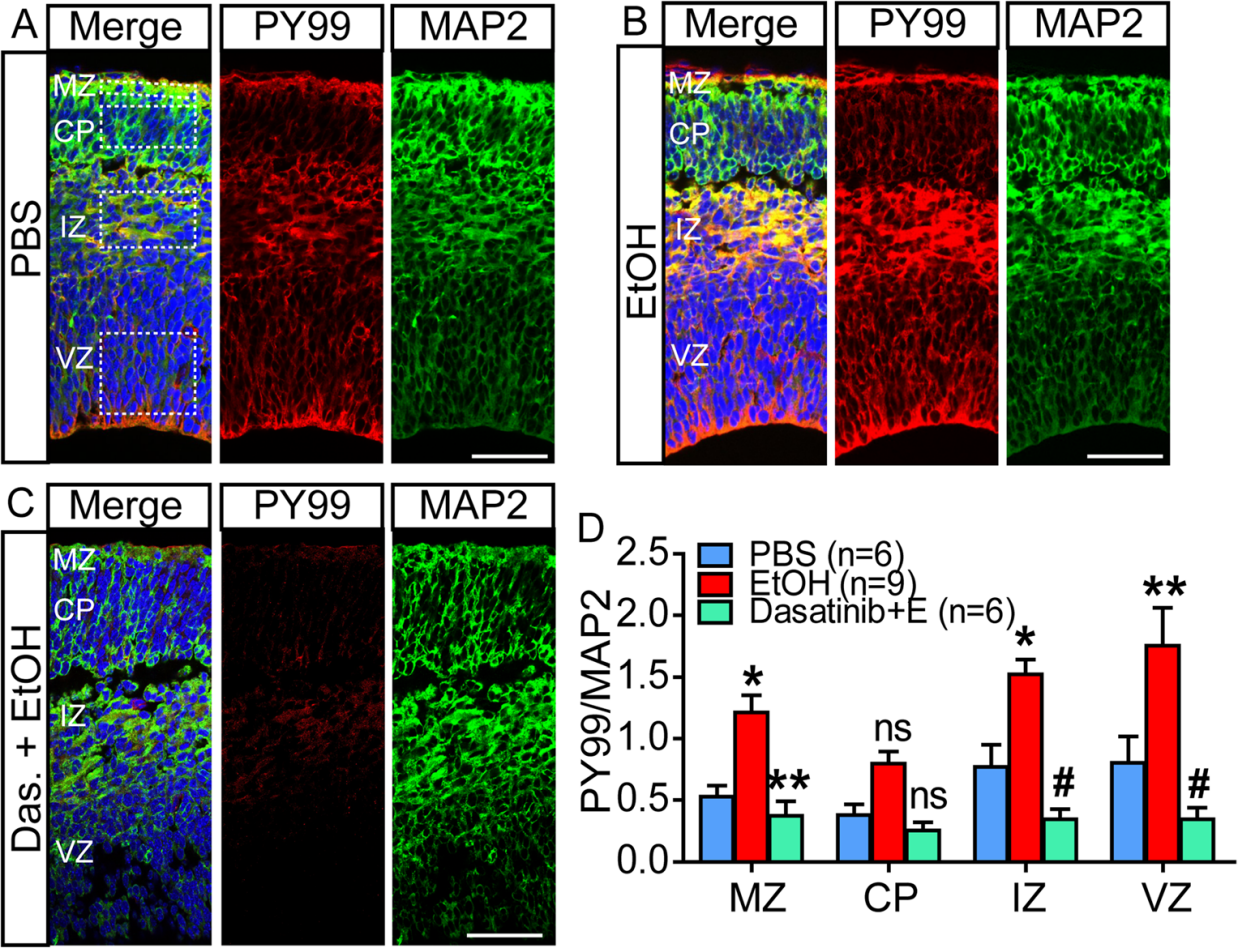
Spatial expression pattern of tyrosine phosphorylation in embryonic cortex 10 min after maternal ethanol exposure with or without dasatinib. Coronal sections of E15 cortex from in utero exposed embryos were immunostained for phosphotyrosine (pY99) and MAP2. In comparison to **A** PBS exposed embryos, **B** ethanol exposed embryos (i.p. 4g /kg) showed significant increases in pY99 content in the VZ, IZ, and MZ. **C** Pretreatment of the dam with 20 mg/kg dasatinib completely blocks the pY99 increase. Scale bar, 100 μm. **D** Densitometric quantification of the immunosignal. MAP2 immunosignal is used as an internal control. Statistical determination by one-way ANOVA followed by Bonferroni’s post hoc tests between groups. *p < 0.05, **p < 0.01, ^#^*p* < 0.001

We used anti-phosphotyrosine (pY99) as well as anti-microtubule-associated protein 2 (MAP2) IHC to determine the spatial and temporal patterns of increased tyrosine phosphorylation in the developing cortex. Drop-fixed hemi-brains were cryosectioned and immunostained for subsequent confocal microscopy. Ethanol caused prominent pY99 signal increases in the apical area of the ventricular zone (VZ), where neural precursors are localized, the intermediate zone (IZ) that contains migrating neurons and developing axonal tracts, the subplate (SP) and marginal zone (MZ) that contain transient populations of neurons critical for early cortical patterning (Fig. 1E–I). Importantly, for this study, the MZ also is the initial projection field for the apical dendrite of most developing excitatory neurons, the cell class responsive to Reelin signaling. Consistent with the western blot analyses, the pY99 IHC signal was elevated for 30 min after maternal exposure. To quantify the regional 10 min response, mean fluorescence intensity from regions of interest (ROIs) located in the MZ, CP (cortical plate), IZ, and ventricular zone and the subventricular zone (VZ/SVZ) were compared with corresponding regions in fetal cortices from PBS exposed dams. As shown in Fig. 2 A and B, ethanol increased pY99 reactivity across the cerebral wall compared to PBS-exposed controls, with a ~ 1.5-fold and higher elevations in the VZ, IZ, and MZ (Fig. 2D). In contrast, MAP2 content was not altered by 10 min of ethanol exposure (not shown). The areas of greatest increase corresponded to areas of highest control pY99 levels, and the response in all areas was blocked by pretreatment of the dam with dasatinib (Fig. 2C and D).

### Areas of Increased Tyrosine Phosphorylation Are Also Areas of Src Kinase Expression

**Fig. 3:**
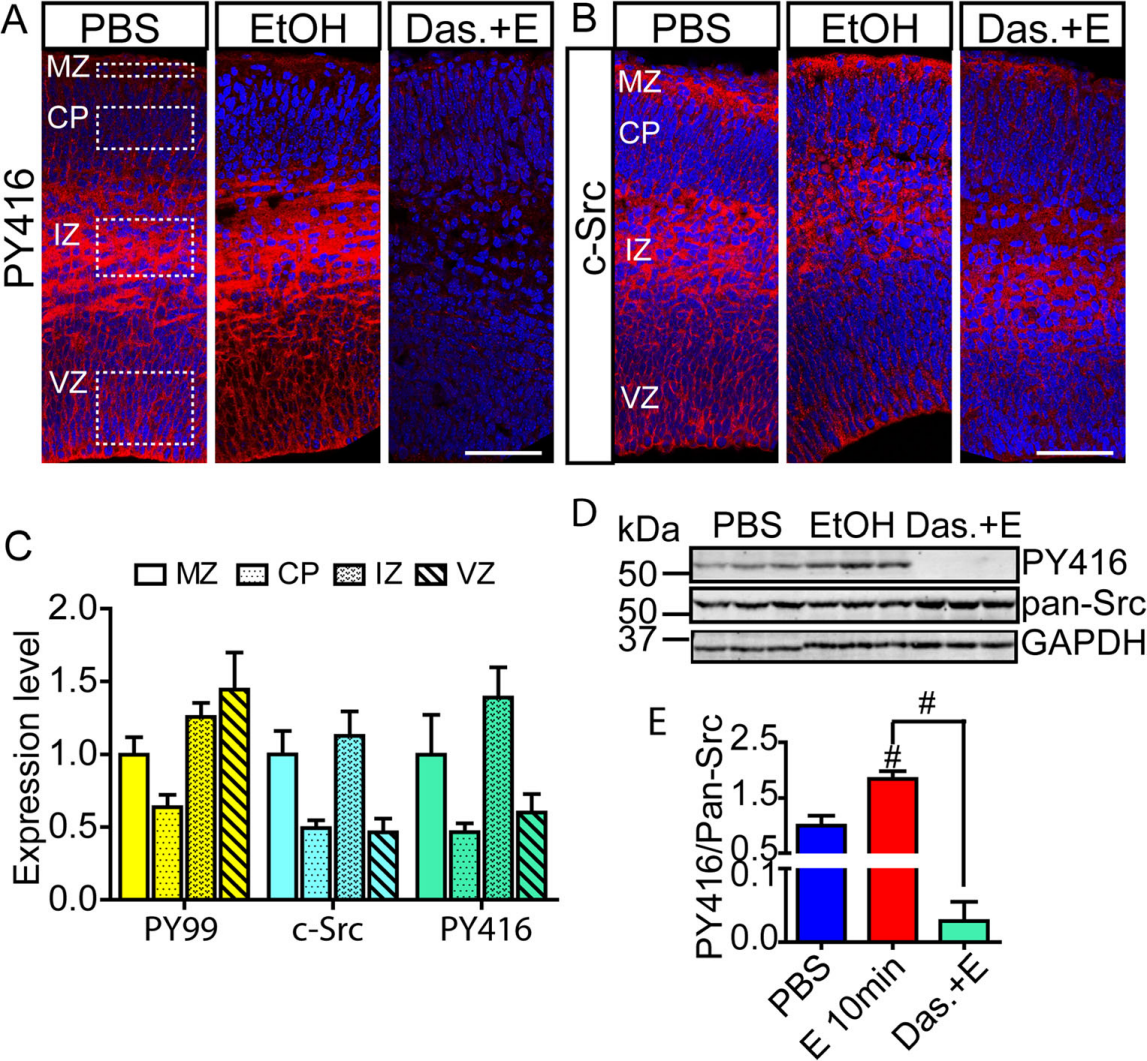
Correspondence between areas of Src activation and pY99 increases after ethanol exposure. **A–B** Representative images of **A** Src activation loop (pY416) and **B** anti-Src immunostaining in E15 cerebral cortex 10 min after intraperitoneal injection of dams with PBS or ethanol (4 g/kg) and with or without dasatinib (20 mg/kg) pretreatment. Scale bar, 100 μm. **C** Mean fluorescence intensity measurements show prominent Src expression and Src activation in the MZ, IZ, and VZ all areas that show significant pY99 response to ethanol. **D** Western blot of cortical lysates probed with pY416, Src, and GAPDH. A significant increase in Src activation loop signal is observed after 10 min ethanol exposure which is completely blocked by dasatinib (20 mg/kg) pretreatment. Samples presented in triplicate. **E** Quantification of blots in (**D**). Statistical determination by one-way ANOVA followed by Bonferroni’s post hoc tests between groups. ^#^*p* < 0.001

In our previous study, 10 min of ethanol exposure caused an increase in activation loop phosphorylation of Src but not the related kinase Fyn, indicating that Src activity is involved in the response [7]. To determine whether the regions of maximal phosphotyrosine response correspond to regions of highest Src expression and Src activation, we performed IHC for total Src and Src pY416 activation loop epitope expression. Total Src and activated Src immunoreactivity was found prominently in the VZ, IZ, and MZ with highest expression in the IZ and low expression in the CP (Fig. 3 A and B). The pY416 signal strongly reduced by pretreatment with dasatinib assayed both by IHC (Fig. 3A) and by western blot (Fig. 3 D and E). The spatial distribution of total Src and active Src immunosignal parallels the spatial distribution of maximal pY99 immunoreactivity (Fig. 3C), consistent with the hypothesis that Src itself is a major contributor to the acute response.

### Migrating Neurons Are Sensitive Targets for Ethanol Exposure

**Fig. 4:**
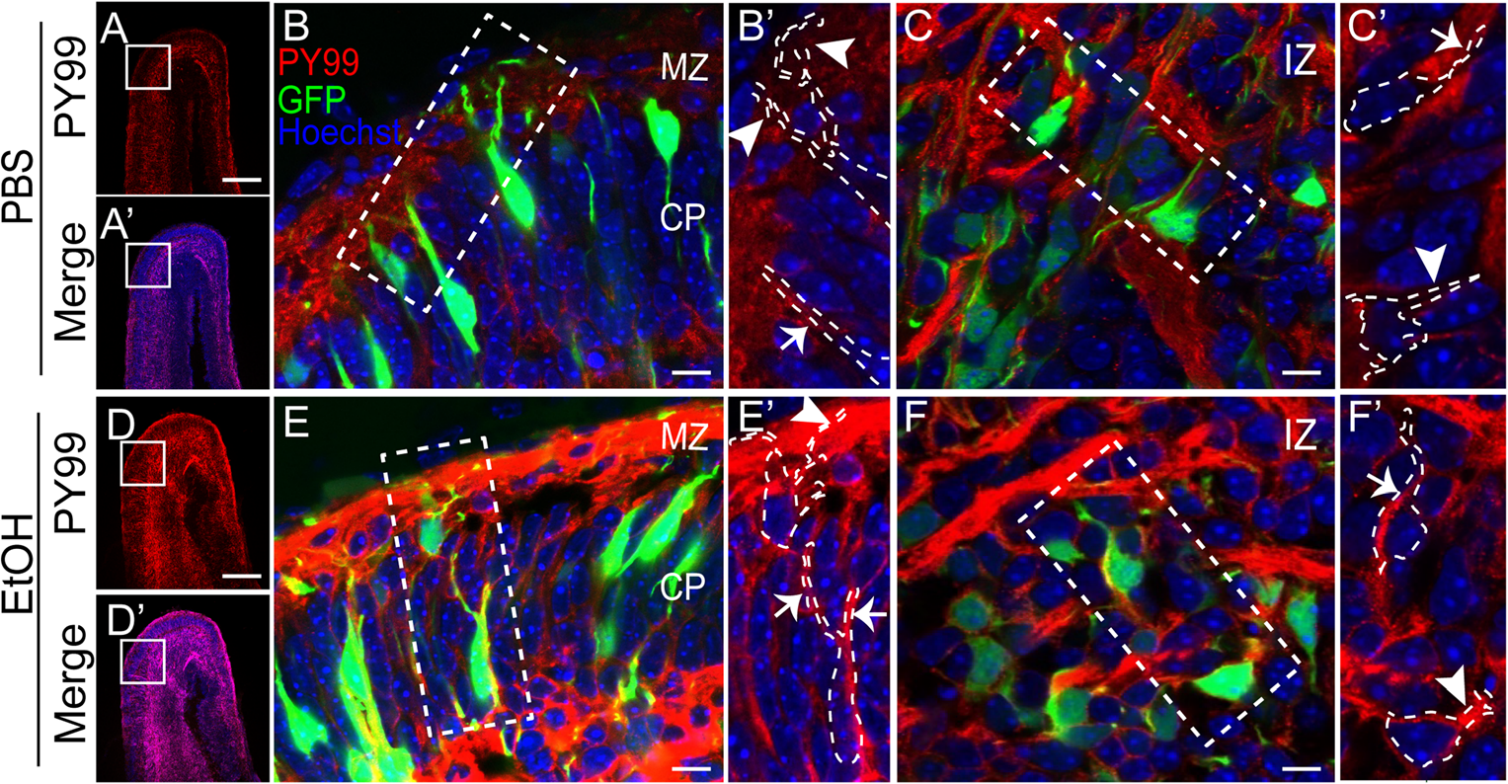
Migrating and differentiating cortical excitatory neurons are sensitive to acute ethanol exposure. Prospective deep layer excitatory neurons were targeted by E13 ex utero electroporation of dorsal neocortex with a GFP expression plasmid. Whole hemisphere explants were cultured on collagen-coated filters for 2 DIV. Explants were then treated with **A–C** PBS or **D–F** 400mg/dL ethanol for 10 min. In comparison to **A** PBS-injected control, significantly higher pY99 immunosignal is observed in **D** ethanol-exposed explants. Higher magnification views of **B,C** PBS and **E,F** ethanol-exposed explants revealed increased pY99 immunosignal in cell bodies and neurites of migrating and differentiating neurons in the CP and multipolar neurons in the IZ. The insets show traced outlines of the GFP^+^ cells and the corresponding pY99 signal in those regions. Sections were counterstained with Hoechst. Scale bar for (**A**) and (**D**) is 100 μm. Scale bar for (**B**), (**C**), (**E**), and (**F**) is 10 μm

To determine the cell classes generating the elevated phosphotyrosine response to ethanol, we used ex utero electroporation of CAG-GFP expression plasmid to label developing cortical cells. Electroporations were performed at E13, and whole hemisphere explants were prepared [30]. After 2 DIV, GFP^+^ cells are found at different stages of migration and development across the cerebral wall. PBS or ethanol (400 mg/dL) was added to the culture medium for 10 min, and explants were immediately drop fixed for subsequent histological analyses. Consistent with our findings from in utero exposed embryos, ethanol-treated explants showed significantly more pY99 signal in the VZ, IZ, and MZ compared to control-treated explants (Fig. 4A–C vs. 4D–F). In addition to the increased pY99 signal in the apical junction of the VZ (not shown), cells with multipolar neuron morphology in the IZ showed enhanced pY99 signal in the periphery of the soma and neurites after ethanol exposure compared to control (Fig. 4 and F). While GFP^+^ somata within the CP showed variable increases in pY99 in response to ethanol, dendrites projecting into the MZ showed a consistent pY99 response (Fig. 4 B and E).

**Fig. 5:**
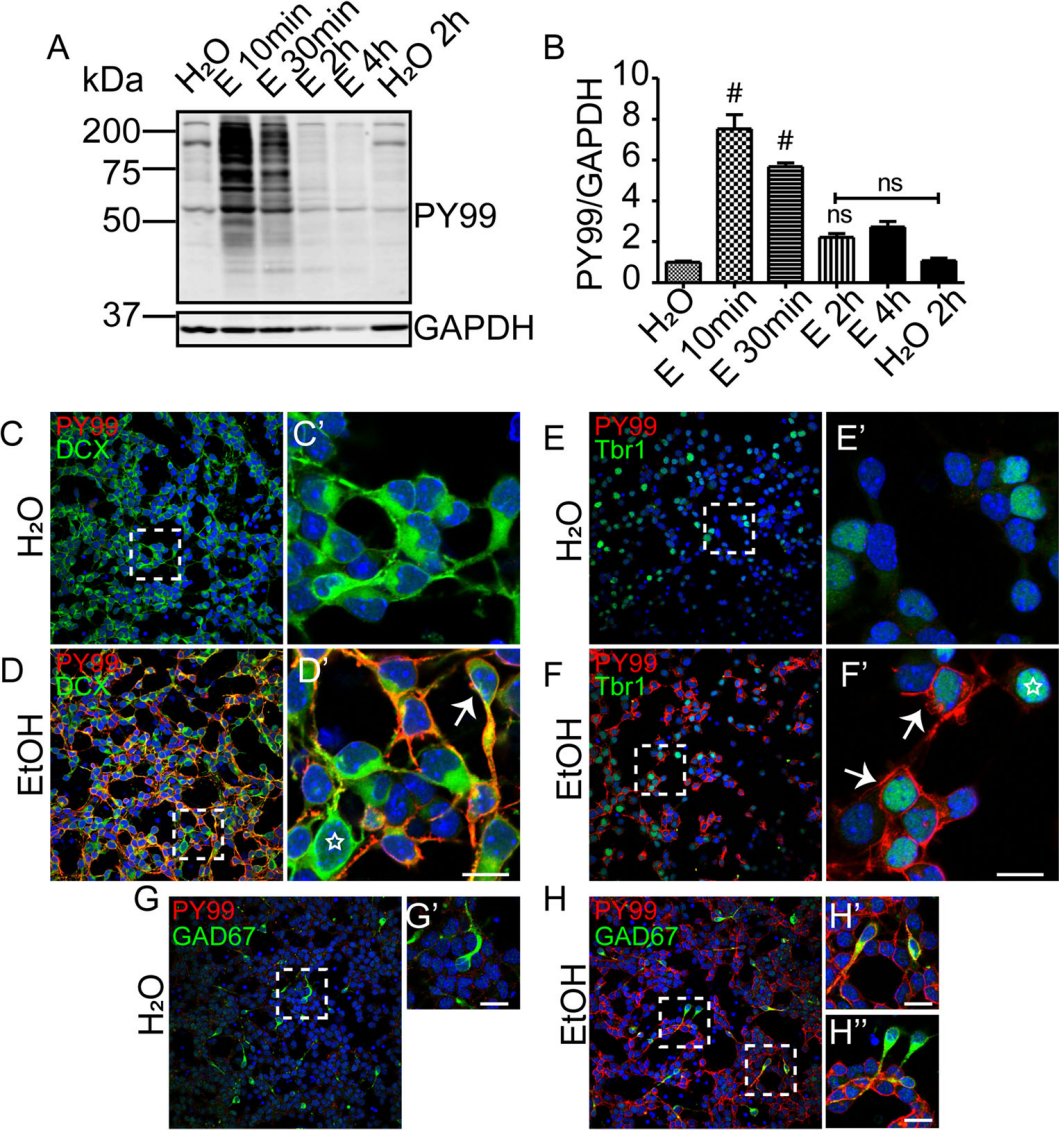
Cultured cortical excitatory neurons and interneurons both respond to ethanol exposure. **A** Characterization of the time course and magnitude of the in vitro tyrosine phosphorylation response in E15 primary cortical culture after ethanol exposure. **B** Densitometric quantification of the response. **C–F** Excitatory neurons respond to ethanol exposure. Primary cortical cultures were fixed and immunolabeled with anti-Doublecortin (Dcx) and pY99 after treatment with **C** H_2_O or **D** ethanol (400mg/dL) for 10 min. At higher magnification (dashed areas), little pY99 signal is observed in control (**C’**), but strong pY99 signal is observed in some (arrow) but not all (star) somata and neurites of Dcx^+^ neurons (an average of 110 Dcx^+^ neurons were counted from 3 experiments) (**D’**). Similarly, **E,F** increased pY99 signal but not all Tbr1^+^ neurons (an average of 80 Tbr1^+^ neurons were counted from 3 experiments) after ethanol exposure. Tbr1 is marker of excitatory cortical neurons at this time in development. **F’** The arrows indicate responding cells. The star indicates a non-responding cell. **G–H** GABAergic neurons respond to ethanol. pY99 immunostaining and GAD67^+^ immunostaining after 10 min of **G** H_2_O or **H** ethanol exposure in E15 primary cortical culture reveal a subset of GAD67^+^ interneurons that are responsive to ethanol (an average of ~ 160 GAD67^+^ cells were counted from 3 experiments). Insets show higher magnification view of boxed regions in (**G**) and (**H**). Scale bar, 10 μm. Statistical determination by one-way ANOVA followed by Bonferroni’s post hoc tests between groups. ^#^*p* < 0.001

To determine the magnitude and time course of the response of primary cultures derived from C57Bl6/J embryos, lysates were prepared at defined times after dosing with ethanol (400 mg/dL) or an equivalent volume of water. Consistent with our prior findings from primary cultures derived from SW embryos [7], a large increase in pY99 signal is observed at 10 min; however, in contrast to SW cultures, the pY99 elevation persists for at least 30 min in cells derived from C57Bl6/J embryos (Fig. 5 A and B). This culture finding is consistent with our finding from in utero dosed embryos and suggests a cell intrinsic difference in the temporal response that depends on strain.

To confirm neurite expression of pY99 in immature excitatory neurons, cortical cultures were stimulated for 10 min with ethanol (400mg/dL) or H_2_O and then fixed and processed for immunocytochemistry. Cortical neurons were immunostained with pY99 and anti-Doublecortin (Dcx), a microtubule associated protein expressed by immature neurons [17] (Fig. 5 C and D), or separately Tbr1, a transcription factor that identifies developing excitatory neurons [31] (Fig. 5 E and F). Ethanol exposure caused a rapid response in pY99 signal on both Dcx^+^ and Tbr1^+^ neurons (arrows) with prominent expression in the perisomatic region and along the neurites. In many neurons, additional signal was observed in cytoplasm and in proximal region of neurites possibly reflecting organelle associated pY. Interestingly, not all neurons (stars) demonstrated a pY99 response, with 62% of Dcx^+^ (n = 110) and 64% of Tbr1^+^ cells (n = 80) exhibiting pronounced pY99 signal.

To determine whether GABAergic interneurons were similarly responsive to ethanol, cultures were prepared from lateral and medial ganglionic eminence tissue to enrich for interneuron populations. After 2 DIV, cultures were treated for 10 min with either ethanol (400 mg /dL) or H_2_O and then fixed and immunostained with GAD67 to identify interneurons and separately pY99 to reveal the phosphorylation response. Ethanol caused a detectable increase in pY99 content in 47% of GAD67^+^ interneurons (Fig. 5 G and H), a figure that is similar to the percentage of Dcx^+^ and Tbr1^+^ cells that show an ethanol response. These findings confirm that developing excitatory and inhibitory neurons are sensitive to the acute effects of ethanol and indicate differential sensitivity or differences in the timing of the ethanol response between cells.

### Reelin Signaling Is Disrupted in Fetal Cortex After Maternal Ethanol Exposure

**Fig. 6:**
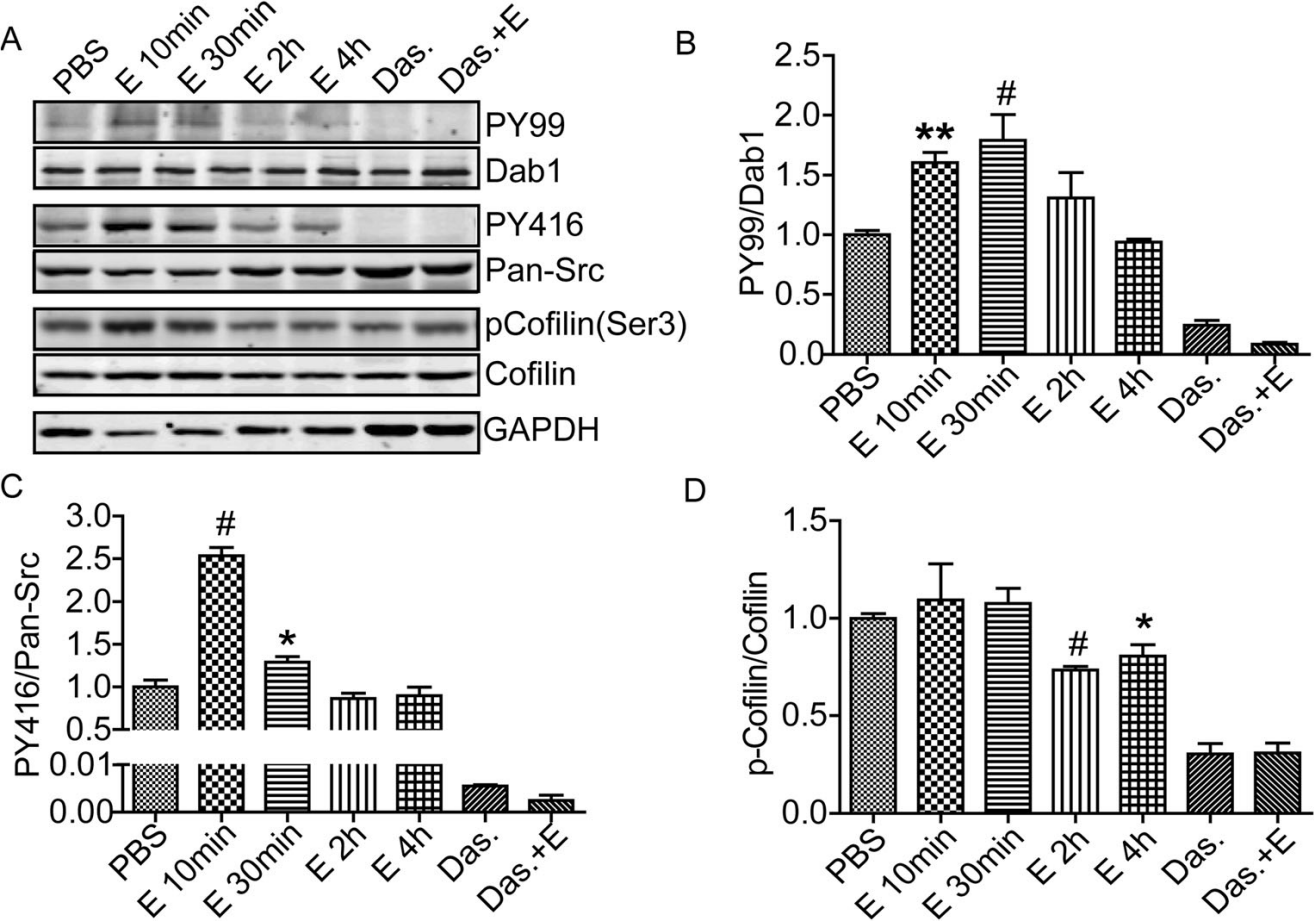
Sustained disruption of Reelin signaling in fetal cortex by maternal ethanol exposure. **A** Western blot detection of tyrosine phosphorylation level of Reelin signaling adaptor protein Dab1, Src activation loop (pY416), and the actin severing protein n-cofilin in E15 embryonic cortical lysate cortex after PBS or 10, 30, 120, and 240 min of maternal ethanol exposure (i.p., 4 g/kg). **B,C,D** Corresponding densitometric quantification of blots reveals a sustained increase in tyrosine phosphorylation of Dab1 (**B**) as well as Src activation loop (pY416) (**C**). Both phosphorylation events are completely blocked by pretreatment of the dam with dasatinib (20 mg/kg). In addition, **D** Ser3 phosphorylation of the actin severing protein n-cofilin starts to decline at 2 h after ethanol exposure. Densitometric values are first normalized the corresponding signal from total protein and then compared to PBS group. One-way ANOVA followed by Bonferroni’s post hoc comparisons tests were used. *p < 0.05, **p < 0.01, ^#^*p* < 0.001

Normally, Reelin binding to its receptors leads to SFK activation, Dab1 tyrosine phosphorylation, and the subsequent phosphorylation of downstream elements including Ser3 on the actin severing protein n-cofilin ([32], a). In vitro, ethanol exposure initially activates Src and downstream tyrosine phosphorylation but ultimately blocks Reelin signaling and causes n-cofilin Ser3 dephosphorylation in vitro [7]. To determine whether these Reelin signaling components are disrupted by in utero exposure, and the time course of that disruption, cortical lysates were prepared from embryos at defined times after maternal injection (i.p. 400 mg/dl). Consistent with our in vitro findings, Src and an 80kD protein, Dab1, show aberrant elevated tyrosine phosphorylation for at least 30 min after exposure (Fig. 6A–C). This indicates that acute ethanol exposure inappropriately activates the Reelin signaling pathway in vivo. As with our prior study, n-cofilin shows aberrant Ser3 dephosphorylation at 4 h after exposure (Fig. 6 A and D) suggesting that acute ethanol exposure may disrupt actin dynamics in the fetal cortex for a 4-h period after maternal exposure. Pharmacological inhibition of SFKs by dasatinib pretreatment alone also caused cofilin Ser3 dephosphorylation suggesting that the response of cofilin after ethanol exposure may possibly represent longer term SFK inhibition by ethanol as we found in vitro [7] but that is not reflected by pY416 status in vivo (Fig. 6 A and C).

### Altered Cytoskeletal Protein Expression and Golgi Complex Morphology After Ethanol Exposure

**Fig. 7:**
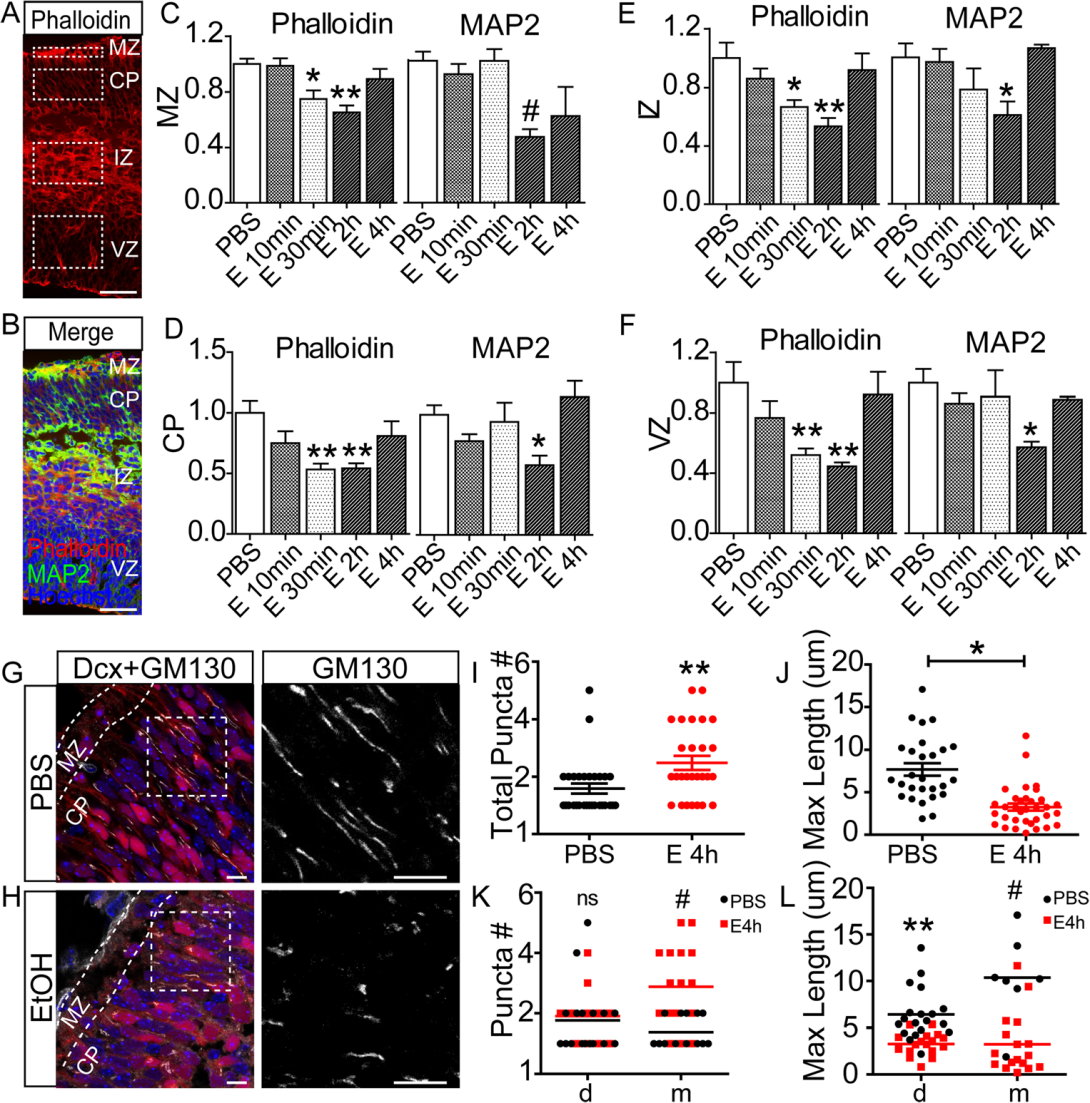
Quantified mean immunofluorescence intensity of phalloidin and MAP2 signals, broken out by region and treatment. **A–B** Alexa555-phalloidin and anti-MAP2 immunostaining in E15 cerebral cortex. Scale bar, 100 μm. **C–F** Significant reduction of the phalloidin signal intensity is observed across the cerebral wall by 30 min after ethanol exposure, whereas MAP2 signal intensity diminishes by 2 h. Signal intensities were quantified from corresponding regions of interest identified in (**A**) (dashed rectangles). **G–H** GM130 immunostaining of E15 cortical sections derived from Dcx-dsRed^14Qlu/J^ transgenic embryos after 4 h of PBS or ethanol exposure. Scale bar, 10 μm. **I–J** Quantitation of total GM130 puncta number (*n* = 29 cells for control and 27 cells for ethanol treated) and the maxim of the length (n = 27 cells for control and 34 cells for ethanol) from cortical plate neurons. **K–L** Quantification of response broken by migratory and postmigratory neuronal morphology. Statistical determination by two-way ANOVA followed by Bonferroni’s post hoc tests between groups. *p < 0.05, **p < 0.01, ^#^*p* < 0.001. Abbreviations: MZ, marginal zone; CP, cortical plate; IZ, intermediate zone; VZ, ventricular zone

Dephosphorylation of Ser3 on n-cofilin is known to increase filamentous-actin (F-actin) severing activity [33, 34] and would be expected to alter F-actin content in the cortex. Our prior studies have shown ethanol-dependent disruption of dendrites and altered expression F-actin, MAP2, and Golgi complex morphology both in culture and in explants [7, 16]. To determine whether similar disruptions occur in vivo after maternal exposure, sections were stained with Alexa 555-phalloidin to detect F-actin and immunostained with anti-MAP2 to detect neurites (Fig. 7 A and B). Consistent with these prior studies, both phalloidin and MAP2 signals showed a pronounced decrease in expression by 2 h. This effect was observed across the cerebral wall including the MZ and IZ, but also the SVZ, an area of active neuronal proliferation (Fig. 7C–F). Interestingly, the difference in phalloidin signal was observed at 30 min of exposure, whereas the difference in MAP2 signal was detected at 2 h suggesting that microtubule cytoskeletal alterations may follow actin disruptions.

The Golgi complex performs multiple essential functions in the developing cortical neurons [35, 36] [37] and can be morphologically and functionally regulated by Src signaling [38]. We and others have shown reduced extension of Golgi apparatus into the apical dendrite after ethanol exposure [16, 39] and after Reelin signaling is disrupted [15, 40–42]. Consistent with these prior studies that employed explants and cell culture models, maternal ethanol exposure also caused sustained (4 h) disruption of Golgi complex morphology in fetal cortical neurons with increased fragmentation and less extension into the apical dendrite (Fig. 7G–L). These findings establish a temporal sequence of ethanol-dependent disruptions and shows that these effects can impact fetal neurons for hours after initial maternal exposure.

## Discussion

In this study, we provide evidence that maternal exposure to ethanol leads to rapid activation of Src family kinases and an elevation of tyrosine phosphorylation in multiple fetal cortical regions and cell types. Importantly, this brief phosphorylation increase is followed by an extended period of at least 2 h in which the Reelin signaling effector n-cofilin is dephosphorylated and F-actin content across the cerebral wall is reduced. In addition, we observed disrupted Golgi apparatus morphology that is similar to that observed with deficiency in Reelin signaling. These findings suggest that ethanol exposure may selectively impact SFK-dependent signaling pathways in the fetal cortex after maternal ethanol exposure.

Our current findings both in vitro and in vivo outline a model in which transient SFK-activation by ethanol initiates a sequence of events that potential could impact signaling for extended periods of time. During the first phase of the ethanol response, Src is activated causing the tyrosine phosphorylation of its own activation loop, and Dab1, an adaptor protein essential for Reelin signaling. Our western blot analyses reveal that activation of SFKs by ethanol causes non-specific phosphorylation of multiple other protein species besides Dab1 at ~ 80 kD and Src itself at ~ 57 kD and this response is distinct from the relatively specific activation of Src and Dab1 by Reelin [43, 44]. Although the identity of these other proteins is currently unknown, this inappropriate tyrosine phosphorylation likely indicates the dysregulation of multiple signaling pathways besides Reelin.

SFKs are essential components of several developmentally important signaling pathways [45] [46] [47]. While mice singly deficient in the SFKs Src and Fyn do not show obvious brain malformations [14], mice singly deficient in Src exhibit small size and osteopetrosis (increased bone density) [48]. In contrast, mice doubly deficient in Src and Fyn show the *reeler* cortical phenotype and profound layering and dendritic disruptions during early cortical development [14, 49]. This suggests some redundancy in SFK signaling but also highlights that inactivation of both SFKs blocks Reelin signaling during early cortical development.

The elevation of phosphotyrosine content by ethanol exposure is relatively brief (5–30 min) and is followed by a tyrosine dephosphorylation phase when total phosphotyrosine levels drop to baseline. During the latter period in vitro, application of Reelin protein could not initiate Reelin signaling, and no increase in Dab1 phosphorylation could be elicited [7]. During this period in vivo, pY416 levels remained at baseline, and since this antibody recognizes both Src and Fyn activation domains, both SFKs may be inactive during the latter phase of ethanol exposure. Inactivation of both SFKs could produce the phenotypic convergence between Reelin deficiency and ethanol exposure. Consistent with a sustained inactivation of Reelin signaling, we found that Ser3 of n-cofilin did drop below baseline indicating a sustained activation of this actin severing protein that is normally inactivated by Reelin signaling [32, 50].

Similarly, we observed disrupted Golgi morphology in the developing apical dendrite after maternal ethanol exposure which paralleled the morphological disruptions observed with Reelin-signaling disruptions [15, 42]. As Golgi and Golgi outposts perform critical functions in developing dendrite including microtubule stabilization, posttranslational protein modification, and transport as well as new membrane production [35, 36, 51] [37, 52–54], this finding may provide insight into some of the sustained disruptions of dendritic function after fetal exposure [55, 56]. Although the SFK-dependent biochemical effects of the acute exposure appear to largely resolve by 4 h post treatment, neuronal damage may be more permanently encoded through sustained changes in Golgi function and neurite outgrowth subsequent to SFK hyperactivation. Moreover, repeated acute exposures such as those that can be found with some forms of alcohol abuse disorder would be expected to cause successive periods of SFK disruption and, potentially, additive disruptions of neuronal development.

Disruption of SFK signaling may also impact neurite growth in other ways besides altering Reelin signaling [57] [45] [46] [47]. Src has important roles in both dendritic orientation and stabilization [58, 59] that may work in concert with Reelin-Dab1 signaling [60] [42] [40]. Neurite outgrowth and navigation is disrupted by ethanol [61] [62] [63] [64], and axonal growth can also depend on Src [47, 65, 66] [67]. Consistent with these findings, we observed that the intermediate zone and marginal zone, a major area of axonal and dendritic development respectively, also shows high Src expression and tyrosine phosphorylation in response to ethanol suggesting particular sensitivity to ethanol exposure.

The mechanism of ethanol-dependent Src activation is not clear: Ethanol activates a closely related SFK (Fyn) by inhibiting a regulatory STEP (striatal enriched phosphatase) [68, 69]. However, this activation of Fyn occurred in striatal neurons and did not involve Src. In contrast, our prior study suggested that Fyn is not strongly activated in primary cortical neurons by ethanol [7]. Moreover, our prior study showed that preincubation with phenylarsine oxide (PAO), a membrane-permeable inhibitor of class I phosphotyrosine phosphatases did not prevent ethanol-induced activation of Src in vitro. This suggests that phosphatase inhibition is unlikely to be the major mechanism of initial Src activation in the present study. Additional studies have found that ethanol may disrupt signaling in cholesterol-enriched lipid rafts [70] [71] including SFK-containing rafts [72]. However, in our prior study, addition of cholesterol nor cholesterol depletion using methyl-β-cyclodextrin did not dramatically alter the Src activation response of cultured neurons to ethanol [7]. Thus, the mechanism of Src activation in embryonic cortex by ethanol remains unclear.

Altered phosphatase activity functioning downstream of Src may be involved in the n-cofilin Ser3 dephosphorylation that is sustained for hours after initial ethanol exposure. While phosphatases are generally thought to be constitutively active and not highly regulated, regulatory mechanisms have been identified [73]. For example, as noted above, ethanol exposure can specifically inactivate STEP phosphatase [69]. There is also evidence that broad forms of cellular stress such as reactive oxygen species and ischemia can cause elevated tyrosine phosphatase activity [73] [74] raising the possibility of elevated phosphatase activity at the later time points after acute ethanol exposure. Slingshot homologs 1–3 (SSH 1–3) have been shown to dephosphorylate cofilin [75], and re-analysis of our prior transcriptional profiling study [76] as well as the Genepaint in situ database (https://gp3.mpg.de) shows that these phosphatases are expressed in the developing mouse cortex at E14.5, raising the possibility that their dysregulation may contribute to the later stage of the acute ethanol response. Alternatively, n-cofilin dephosphorylation may be due to the inhibition of kinases such as LIMK1 which phosphorylate n-cofilin at Ser3 [77]. N-cofilin activity may be critical for both dendritic outgrowth [78] and to maintain Golgi morphology via a RhoA signaling pathway involving LIMK1, cofilin, and slingshot [79]. Understanding the balance of phosphatase and kinase activity during the persistent stage of the acute ethanol response will be critical to understanding the mechanisms of ethanol-induced signal suppression and may provide insight into the disrupted connectivity associated with fetal ethanol exposure.

Ethanol exposure causes broad disruptions to the developing nervous system [3–6], and explaining the diversity of ethanol’s effects remains an important goal in fetal alcohol syndrome disorder (FASD) studies. This study raises the possibility that dysregulated Src activity may be an early response to acute ethanol exposure and may be critical to understanding how ethanol disrupts multiple signaling pathways in the fetal brain. This work should enable further mapping of brain region and cell-type sensitivities to exposure as well as guide future studies of the genetic susceptibilities to ethanol-induced neural damage.

## Supplementary Information

Supplementary Figure. Time course of maternal blood ethanol concentrations (BEC) and fetal amniotic fluid concentrations (AEC) over 4 h following ethanol exposure at E15 (PNG 747 kb)

High Resolution Image (TIF 277 kb)

## Data Availability

All authors agree that all data and materials as well as software application comply with field standards.
