# Supplementary figures and images for: Maternal Ethanol Exposure Acutely Elevates Src Family Kinase Activity in the Fetal Cortex
Source: Mol Neurobiol. 2021 Jul 16;58(10):5210–23. doi: 10.1007/s12035-021-02467-x (PMC8497457; doi:10.1007/s12035-021-02467-x)

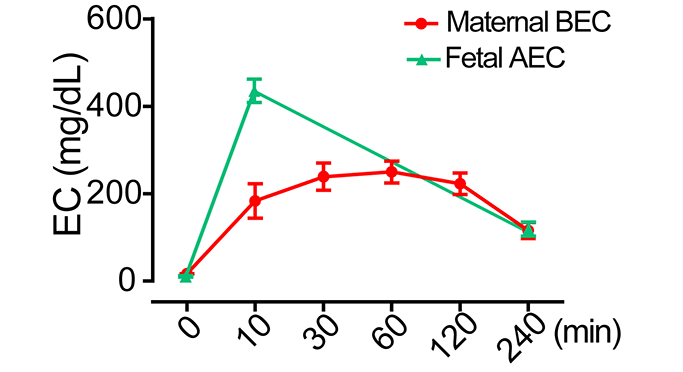

Supplement: Supplementary file 1 — Supplementary Figure. Time course of maternal blood ethanol concentrations (BEC) and fetal amniotic fluid concentrations (AEC) over 4 h following ethanol exposure at E15 (PNG 747 kb) [file 12035_2021_2467_Fig8_ESM.png]

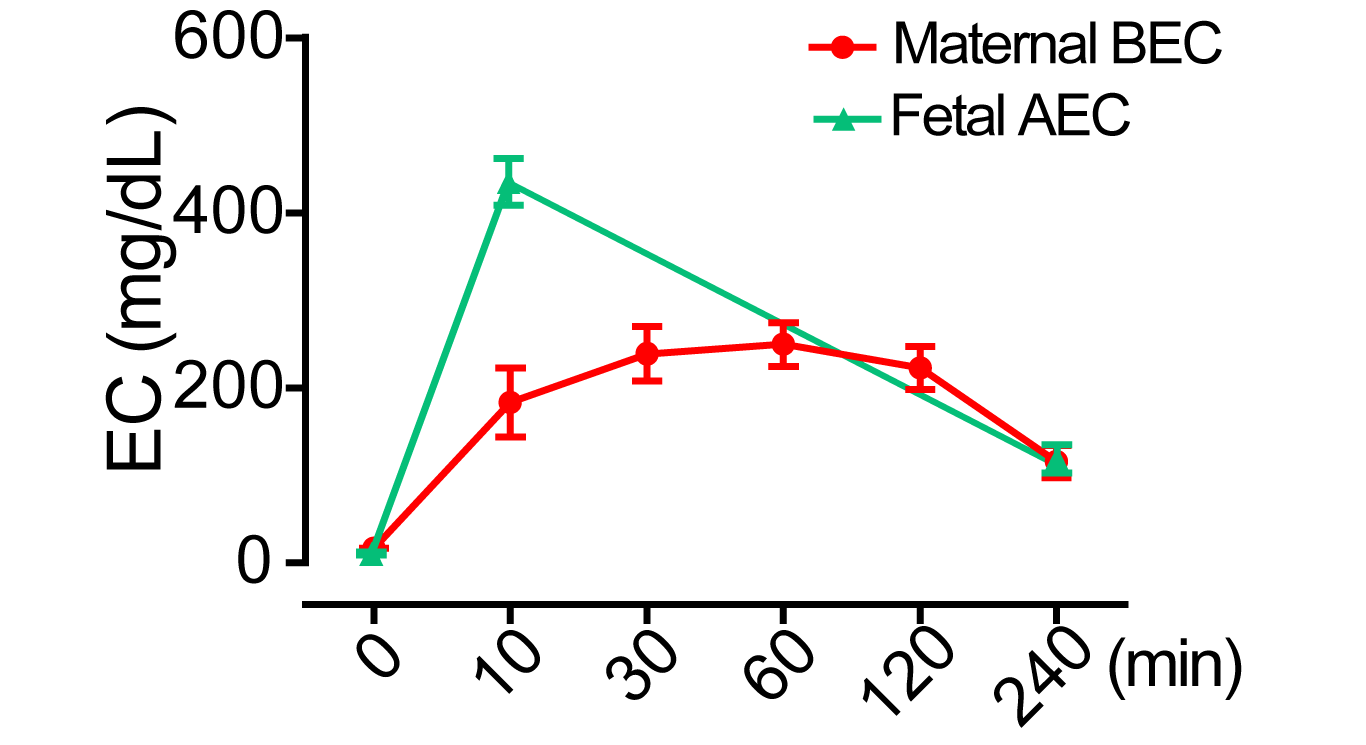

Supplement: Supplementary file 2 — High Resolution Image (TIF 277 kb) [file 12035_2021_2467_MOESM1_ESM.tif]
